# Functional results of endoscopic arytenoid abduction lateropexy for bilateral vocal fold palsy

**DOI:** 10.1007/s00405-021-07199-1

**Published:** 2021-12-02

**Authors:** László Rovó, Vera Matievics, Balázs Sztanó, László Szakács, Dóra Pálinkó, Christopher T. Wootten, Péter Pfiszterer, Zoltán Tóbiás, Ádám Bach

**Affiliations:** 1 Department of Otorhinolaryngology-Head and Neck Surgery, Faculty of Medicine, University of Szeged, Tisza Lajos krt. 111, Szeged, 6725 Hungary; 2 Department of Otolaryngology-Head and Neck Surgery, Vanderbilt University Medical Center, Nashville, TN USA

**Keywords:** Bilateral vocal fold palsy, Endoscopic arytenoid abduction lateropexy, Minimally invasive surgery, Voice quality

## Abstract

**Purpose:**

Endoscopic arytenoid abduction lateropexy (EAAL) is a reliable surgical solution for the minimally invasive treatment of bilateral vocal fold palsy (BVFP), providing a stable airway by the lateralization of the arytenoid cartilages with a simple suture. The nondestructive manner of the intervention theoretically leads to higher regeneration potential, thus better voice quality. The study aimed to investigate the respiratory and phonatory outcomes of this treatment concept.

**Methods:**

61 BVFP patients with significant dyspnea associated with thyroid/parathyroid surgery were treated by unilateral EAAL. Jitter, Shimmer, Harmonics to Noise Ratio, Maximum Phonation Time, Fundamental frequency, Voice Handicap Index, Dysphonia Severity Index, Friedrich’s Dysphonia Index, Global-Roughness-Breathiness scale, Quality of Life, and Peak Inspiratory Flow were evaluated 18 months after EAAL.

**Results:**

All patients had a stable and adequate airway during the follow-up. Ten patients (16.4%) experienced complete bilateral motion recovery with objective acoustic parameters in the physiological ranges. Most functional results of the 13 patients (21.3%) with unilateral recovery also reached the normal values. Fifteen patients (24.6%) had unilateral adduction recovery only, with slightly impaired voice quality. Eleven patients (18.0%) had false vocal fold phonation with socially acceptable voice. In 12 patients (19.7%) no significant motion recovery was detected on the glottic level.

**Conclusion:**

EAAL does not interfere with the potential regeneration process and meets the most important phoniatric requirements while guaranteeing the reversibility of the procedure—therefore serving patients with transient palsy. Further, a socially acceptable voice quality and an adequate airway are ensured even in cases of permanent bilateral vocal fold paralysis.

## Introduction

Despite the continuous development of surgical techniques, bilateral recurrent laryngeal nerve (RLN) injury mainly occurs as a major complication of thyroid/parathyroid surgery [1]. The position of the vocal folds, general health, cardiopulmonary reserve, and physical activity of a patient mainly determine the severity of dyspnea caused by consequential bilateral vocal fold palsy (BVFP). Life-threatening dyspnea obviously requires urgent surgical intervention to prevent acute asphyxiation. Moreover, a patient’s quality of life may be adversely affected by mild dyspnea as well; therefore, restoration of adequate airway plays a crucial role in these cases too [2]. Tracheostomy has been accepted for centuries as the gold standard to achieve definitive treatment, despite its potentially severe psychological and somatic adverse effects [3–5]. Continuous improvement of anesthesia and the development of diagnostic and surgical techniques over recent decades have enabled physicians to avoid implementing this highly unpleasant intervention. However, most surgical techniques employ resection of laryngeal structures on some level (e.g., arytenoidectomy and/or cordotomy) having a significant negative impact on voice quality [2, 6–8]. Accordingly, these ablative procedures are generally applied when permanent paralysis is verified by laryngeal electromyography as well [9, 10].

Another surgical approach has arisen since the beginning of the twenty-first century: displacing the vocal fold without tissue resection [7]. These mainly endoscopic procedures achieve vocal fold lateralization using a simple suture. These potentially reversible techniques provide permanent airway restoration in BVFP as well [11, 12]. Although this concept promises simpler management of BVFP, the precise roles of lateralization techniques are still undefined. Further phoniatric studies have yet to evaluate the potential benefits of these techniques, particularly compared with other glottis enlargement procedures.

Endoscopic arytenoid abduction lateropexy (EAAL) is a minimally invasive, quick, and safe intervention for the treatment of vocal fold palsy, that provides an immediate patent airway [13–15]. Via EAAL the arytenoid cartilage can be stabilized in its maximally abducted position in a physiological manner through endoscopically inserted sutures, without resection of delicate phonatory structures. The Endoscopic Thread Guide Instrument (ETGI; Mega Kft, Szeged, Hungary) is designed for accurate, fast and safe suture loop creation for this surgical procedure [13]. The functional results of this surgical technique are reported hereby from the phoniatric and spirometric point of view in cases of temporary and permanent bilateral vocal fold palsy.

## Materials and methods

### Patients

Between January 2017 and December 2018, we sequentially enrolled patients with BVFP associated with thyroid or parathyroid surgery. Unilateral EAAL served as the treatment of choice in all cases to manage their severe/moderate dyspnea. Fifteen patients with thyroid cancer underwent additional radioiodine therapy. Since I-131 therapy does not affect voice quality, the study-group could be considered homogenous from this perspective [16, 17]. The status of the glottis (particularly the mobility of the vocal folds) was evaluated using a 70° rigid endoscope during regular control examinations of patients under local anesthesia. The patients were categorized into well-defined groups according to the recovery of vocal fold motion as follows:

- *Group I*: patients with bilateral complete motion recovery.
- *Group II*: patients with complete unilateral vocal fold motion recovery.
- *Group III*: patients with partial vocal fold motion recovery.
- *Group IV:* patients with false vocal fold phonation without significant active glottic movement.
- *Group V*: patients without significant motion recovery.

### Surgical procedure

**Fig. 1:**
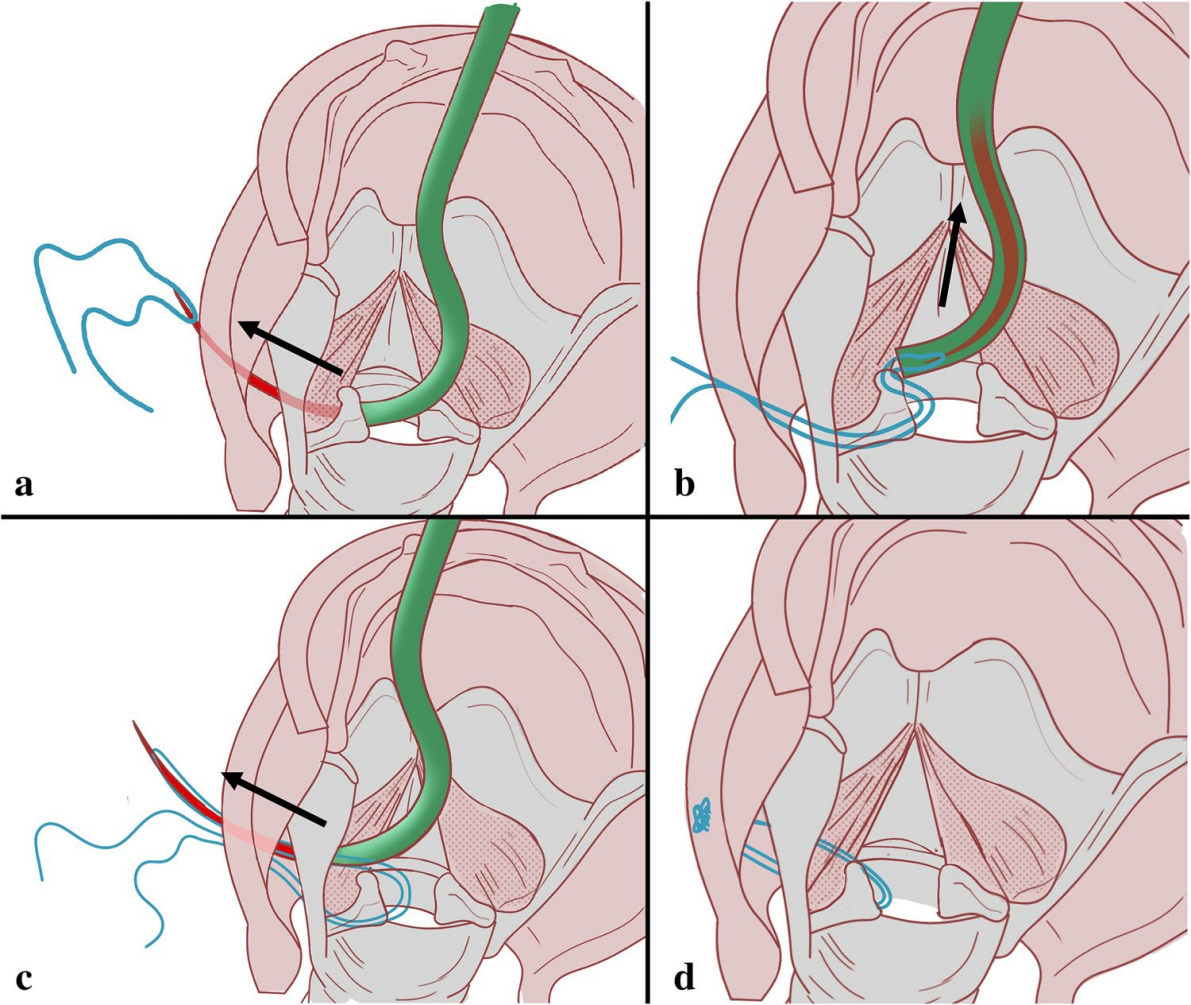
Left-sided endoscopic arytenoid abduction lateropexy. (schematic drawing, posterior view of the larynx, the arrow shows the movement of the blade). **a** The built-in, curved blade is pushed through under the vocal process out to the surface of the neck, and a nonabsorbable suture thread is laced through the hole at the tip of the blade. **b** The doubled-over thread is pulled back with the blade, into the laryngeal cavity. **c** After a repeated tilting of the arytenoid cartilage, the blade (and the thread) is pushed out above the vocal process to the outer surface of the neck. **d** The arytenoid cartilage is stabilized in its maximally abducted position in a physiological manner

BVFP patients underwent unilateral endoscopic arytenoid abduction lateropexy as described in our earlier publications [13–15]. EAAL was performed under general anesthesia via total intravenous anesthesia and supraglottic jet ventilation. The glottis was explored using a Weerda laryngoscope, and the mobility of the cricoarytenoid joints was bluntly checked to exclude mechanical fixation. The laryngeal mucosa was subsequently disinfected and then the arytenoid cartilage was tilted backward and upward using the tip of the ETGI. The curved, built-in blade was pushed through under the vocal process out to the surface of the neck. During this step, the assistant surgeon led a nonabsorbable suture thread (Prolene 1.0; Ethicon, Somerville, NJ) through the hole at the tip of the blade (Fig. 1a). The blade was then retracted into the laryngeal cavity with the doubled-over thread (Fig. 1b). The arytenoid cartilage was tilted again, and the blade (with the thread) was again extruded above the vocal process to the outer surface of the neck (Fig. 1c). The double-folded thread was cut by the assistant, the blade was retracted into the laryngeal cavity, and the ETGI was finally removed. The threads were transposed under the skin (on the surface of the sternohyoid muscle) into one of the previous incisions, and the corresponding ends were knotted (Fig. 1d).

In cases of appropriate abduction motion recovery (*Groups I and II*) with sufficient respiratory function, the lateralizing sutures can be removed under local anesthesia. After localization (by palpation) of the suture, an approximately 1-cm-long incision is made in the neck. The sutures are cut, the threads are simply removed from the laryngeal lumen, and the incision is closed.

### Evaluation of functional results

Voice assessment was performed 18 months after EAAL, according to our previously published institutional protocol, which was elaborated by the guidelines of the Committee on Phoniatrics of the European Laryngological Society [18, 19]. The acoustic data were acquired from recordings of three samples of sustained /a:/ at a comfortable pitch and loudness, and a standardized, connected speech sample. Jitter %, Shimmer %, Mean Pitch, Harmonics to Noise Ratio (HNR), and Maximum Phonation Times (MPT) were assessed using Praat 6.1.09 software [www.praat.org]. Peak inspiratory flow (PIF) was measured to objectively evaluate respiratory function [20].

As a component of our subjective evaluation, a jury of three physicians and one speech therapist perceptually analyzed the connected speech recordings according to the G (global) R (roughness) B (breathiness) criteria (GRBAS) scale [21]. The Dysphonia Severity Index (DSI) [22] and Friedrich’s Dysphonia Index (FDI) [23] were also used as complex, calculated linear indexes to evaluate dysphonia. The Hungarian version of the Voice Handicap Index (VHI) was applied for subjective self-evaluation [24]. The functional outcomes of the surgery in terms of breathing, voice, swallowing, and overall satisfaction were assessed using a Quality of Life questionnaire [25].

## Results

**Fig. 2:**
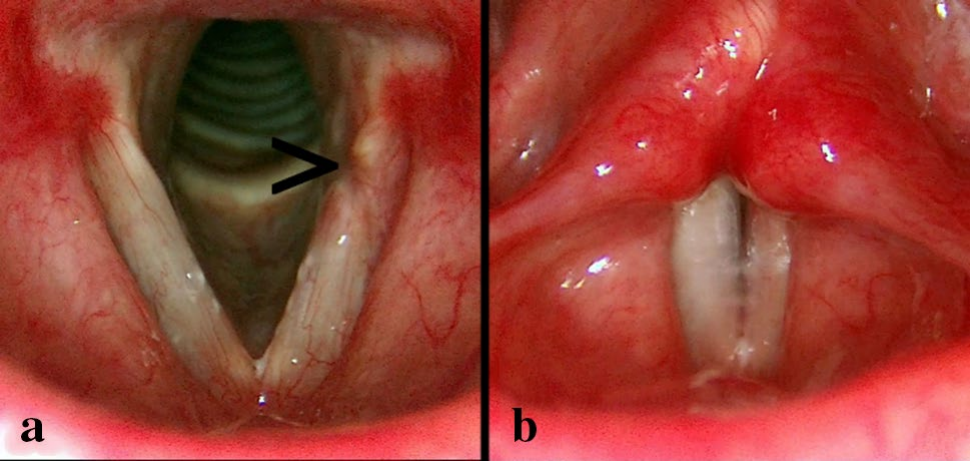
Endoscopic pictures of a 42-year-old female BVFP patient. In the 8th postoperative month, the left-sided lateralizing sutures were removed due to complete bilateral motion recovery. **a** Inspiration; >  = small mucosal impression at the site of the removed lateralizing suture. **b** Phonation

**Fig. 3:**
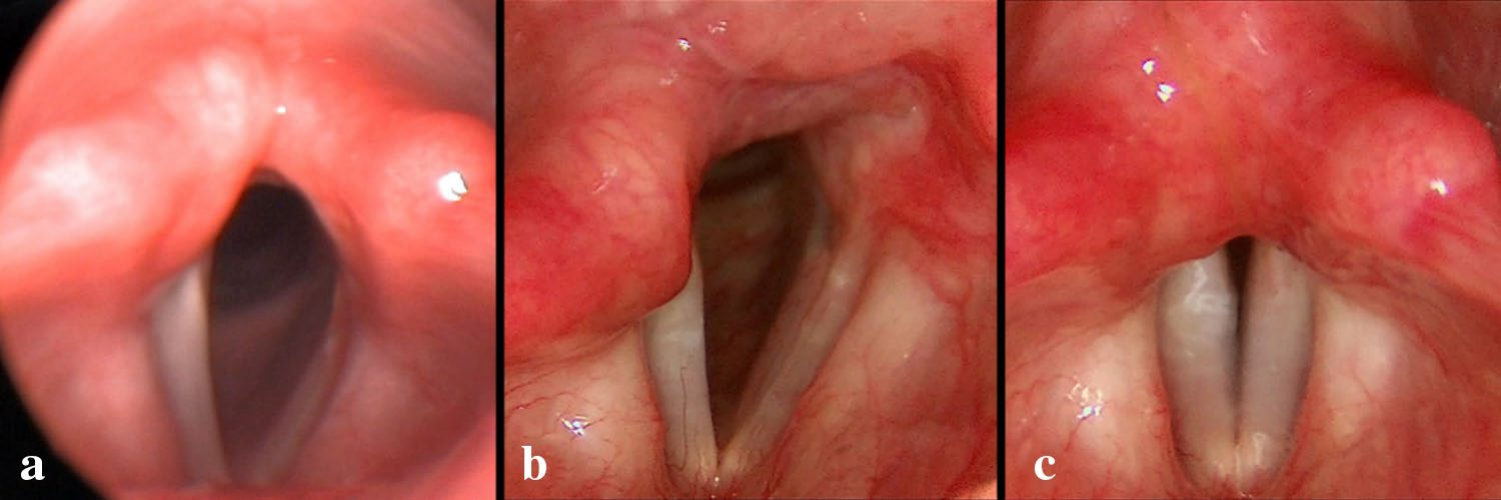
Endoscopic pictures of a 27-year-old female BVFP patient. **a** 2 weeks after EAAL on the left side. No significant vocal fold movements were observed. **b**, **c** Complete motion recovery of the left (lateralized) vocal fold. In the 11th postoperative month, the lateralizing sutures were removed. The contralateral fold remained immobile. **b** Inspiration; **c** phonation

We sequentially evaluated 65 patients who were managed for BVFP. Among them, 4 opted out of the study. (Two patients refused the control examinations; two patients died of cardiac arrest.) The remaining 61 patients (49 females [80.3%] and 12 males [19.7%]; mean age 52.3 years, range 24–82 years) underwent phoniatric assessment. Forty-two patients (68.8%) underwent thyroid surgery 1 day–6 months before EAAL; 19 patients (31.2%) underwent the procedure more than 6 months (range 6–36 months) before airway surgery. Five patients were referred to our department intubated, 7 with a tracheostomy and 1 with acute, life-threatening dyspnea. Definitive extubation/decannulation was successful in these cases. In total, all patients had stable and adequate airway during the follow-up as demonstrated in Table 1. Patients did not report swallowing difficulties or aspiration, and none of them required the use of a nasogastric feeding tube. EAAL associated aspiration pneumonia was not diagnosed in the cohort. Accordingly, no sutures were removed for these reasons. Acute laryngitis and/or suture-related inflammation of the neck were registered in 4 patients. (These events occurred in the 3rd–6th postoperative months.) Repeated EAAL was required for two patients because of spontaneous, partial remedialization of the lateralized vocal fold. Patients’ postoperative PIF improvement enabled them to continue their premorbid daily routines and physical activities. 26 of 61 patients underwent speech therapy sessions after EAAL. The detailed data for voice assessments are presented in Table 2 and are summarized as follows:

- *Group I*: 10 patients (9 females and 1 male) demonstrated *bilateral complete motion recovery* (Fig. 2). Among them, the lateralizing sutures were removed during the second through fourteenth (average 5.4 months) postoperative months. The values of relevant objective aerodynamic and acoustic parameters (MPT, HNR, Jitter, Shimmer) were within their physiological ranges, which correlated with the results of perceptual voice analysis and the average score of VHI (12 points). However, DSI (1.42) and FDI (1.08) revealed detectable voice impairment.
- *Group II*: 13 patients (9 females and 4 males) showed *only unilateral complete vocal fold motion recovery*. After undergoing EAAL, four patients regained complete activity on the lateralized side, and nine patients’ vocal fold regained complete activity on the contralateral side (Fig. 3). The lateralizing sutures of ten patients were removed after 2–6 months (average 3.8 months), although three patients declined removal. Jitter (0.95%), Shimmer (3.73%), and MPT (16.05 s) were within their physiological ranges, and HNR was moderately impaired (13.74 dB). Perceptual analysis and VHI (23.85) demonstrated mild dysphonia. DSI (1.43) and FDI (1.08) showed significant deterioration compared with *group I*.
- G*roup III:* 15 patients (13 females, 2 males) experienced partial vocal fold motion recovery with predominantly adduction improvement. The value of Shimmer (6.45%) was particularly higher compared with previous groups. HNR (14.61 dB) and Jitter (1.28%) were similar to those of *group II*, despite partial (mainly adduction) regeneration. Perceptual grading, DSI (0.63) and FDI (1.60), indicated deterioration of voice quality, which was not fully reflected by the subjective VHI results (28.40).
- *Group IV*: 11 patients (8 females and 3 males) experienced false vocal fold phonation without significant active glottic movement. Accordingly, the objective acoustic parameters significantly decreased compared with those of *groups I–III*, with an average MPT of 4.32 s. At the same time, subjective parameters, particularly VHI (48.18), revealed poor voice quality.
- *Group V:* 12 patients (10 females and 2 males) failed to demonstrate significant motion recovery. Thus, these patients were diagnosed with permanent and complete paralysis. Further deterioration was observed, according to objective and subjective parameters (Table 2).

**Table 1:** Long-term postoperative spirometric results

	Preoperative PIF [l/s]	Late postoperative PIF [l/s]
Number of patients	48*	61
Temporary palsies (Groups I–IV)	1.65 ± 0.41	2.83 ± 0.72
Permanent palsies (Group V)		2.42 ± 0.40

Late postoperative measurement = 18th month after EAAL

*PIF* Peak Inspiratory Flow

*13 patients cannot be measured because of tracheostomy, life-threatening dyspnea or intubation

**Table 2:** Voice assessment of BVFP patients underwent unilateral EAAL and experienced varying degrees of laryngeal motion recovery

	Aerodynamics	Acoustics				Perception				Dysphonia Index	
	MPT [s]	Pitch [Hz]	Jitt [%]	Shim [%]	HNR [dB]	VHI	G	R	B	DSI	FDI
Physiological Values	> 15		< 1.04	< 3.81	> 20	0–120	0–3	0–3	0–3	(− 5)–(5)	0–3
Group I: patients with bilateral complete vocal fold motion recovery (*n* = 10; 9 females and 1 male)											
Mean	18.09	230.31	0.73	2.49	23.42	9.0	0.50	0.3	0.2	3.43	0.60
SD ( ±)	4.58	86.11	0.11	0.47	3.96	3.80	0.52	0.48	0.42	1.38	0.20
Group II: patients with unilateral vocal fold motion recovery (*n* = 13; 9 females and 4 males)											
Mean	16.05	178.09	0.95	3.73	13.74	23.85	1.23	1.23	1.15	1.42	1.08
SD ( ±)	5.26	75.41	0.37	0.67	4.52	8.30	1.17	1.09	0.89	0.44	0.23
Group III: patients with partial vocal fold motion recovery (*n* = 15; 12 females and 3 males)											
Mean	7.16	200.20	1.28	6.45	14.61	28.40	2.20	1.80	2.00	0.63	1.60
SD ( ±)	3.62	66.11	0.21	2.87	3.48	9.87	0.86	0.94	0.85	0.98	0.27
Group IV: patients with false vocal fold phonation (*n* = 11; 8 females and 3 males)											
Mean	4.32	196.19	2.15	7.02	12.62	48.18	2.64	2.00	2.64	− 1.48	1.82
SD ( ±)	1.24	55.31	0.57	4.07	2.49	12.45	0.50	0.89	0.50	0.78	0.76
Group V: patients with no significant motion recovery (*n* = 12; 10 females and 2 males)											
Mean	3.10	177.23	3.14	12.16	9.37	72.17	2.75	2.17	2.75	− 2.33	2.33
SD ( ±)	1.11	27.96	2.39	7.22	6.41	24.17	0.45	0.83	0.45	1.22	0.66

*MPT* maximum phonation time, *Jitt* Jitter, *Shim* Shimmer, *HNR* harmonic to noise ratio, *VHI* Voice Handicap Index, *G* global, *R* roughness, *B* breathiness (from the GRBAS scale), *DSI* Dysphonia Severity Index, *FDI* Friedrich’s Dysphonia Index

## Discussion

The comparison of the functional outcomes of different surgical techniques addressing BVFP raises a complex question, because the functional results depend on the surgical method as well as a patient’s age, sex, mental and physical health, and the potential regeneration of the recurrent laryngeal nerve. Despite numerous studies of humans and animals, insufficient data are available to unambiguously define the pathophysiology of BVFP [26–28]. The intraoperative stretching, mild thermal damage, etc. often cause only axono- or neuropraxy which explains the frequently reported laryngeal function regeneration [1]. However, the exact characterization of functional recovery is rarely cited in the publications connected to the topic of postoperative voice quality [2, 26–30].

The mechanisms of neural regeneration are complex and highly variable. For example, physiological reinnervation, pathological/synkinetic reinnervation, and definitive denervation simultaneously occur to varying degrees [28]. These neurological processes may occur several months after the nerve injury, and the definitive functional results may vary from complete, or nearly complete, vocal fold motion recovery to different types of synkinesis, and less frequently to complete neurological immobility. Moreover, nerve anastomoses, which are individual in human larynges or develop pathologically during healing, may exert supplementary motor effects on the glottic and supraglottic structures [26, 27, 34, 35]. Thus, the requirement for preserving the intrinsic laryngeal muscles during glottis-widening procedures is definitely advantageous even in cases of severe RLN injury because these anatomical structures significantly contribute to residual vocal fold motions and increase the tension of the vocal folds.

The variability of functional recovery indicates that exact categorization of patients according to the result of vocal fold motion recovery is a crucial aspect of the phoniatric evaluation of different glottis-widening techniques. This variability may represent a factor as important as the surgical procedure in determining a patient's postoperative voice. Accordingly, numerous patients with BVFP must be enrolled to create properly powered study groups, which explains the occasionally weak association between voice parameters, even in well-designed surveys [2].

In our present series, 19 of 61 patients (31.1%), or 33 of 122 vocal folds (27.0%) showed complete vocal fold motion recovery. Ten patients experienced bilateral recovery with good phonatory closure. These complete motion recoveries were consistently reflected by objective aerodynamic and acoustic parameters. Perceptual voice analysis and calculated indexes were slightly deteriorated, however, thyroidectomies without RLN injuries can have similar impact on voice quality [36]. This anomaly may be explained by the imperfect regeneration of the fine-tuning mechanisms of voluntary vocal fold motions. In total, 19 nonlateralized vocal folds showed complete recovery of motion (complete abduction and adduction). Meanwhile, the same degree of motion recovery was observed in 14 cases on the side of arytenoid lateropexy. This relatively high rate of regeneration of the lateralized vocal folds confirms the noninvasive and consequently reversible aspect of EAAL. It is particularly important to consider that the lateralizing suture was always placed on the side that was assumed to suffer a more severe RLN injury because of previous thyroid/parathyroid surgery. In cases of complete motion recovery of the lateralized vocal fold only, good glottic closure was observed after the suture was removed. If motion regeneration occurred on the nonlateralized side, the “released” paretic vocal fold remedialized after the removal of the suture [37]. Accordingly, the objective functional results of patients in *Group II* nearly reflected physiological values and correlated with the outcomes of patients with unilateral vocal fold palsy who do not undergo glottic surgery [38, 39]. In our experience, the active and passive mobility of the cricoarytenoid joint remains intact after EAAL. After the removal of the lateralizing suture, no cricoarytenoid joint immobility was observed [40, 41].

Nondynamic parameters of the vocal fold are also critical regarding the voice quality. Such static parameters include the mass, length, and elasticity of the vocal fold [42]. Isshiki et al. (1978) claimed that chaotic vibration patterns and consequential hoarseness can be explained by these unbalanced vocal fold parameters—apart from the inadequate glottic closure. As a minimally invasive procedure, EAAL does not require any resection of the glottic structures. Moreover, the possibility of muscular atrophy is significantly lower because of potential reinnervation. These features of EAAL ensure preservation of the mass of the vocal fold. In addition, EAAL does not damage the membranous part of the vocal fold either. This subregion is principally involved in vibration, and when scarred, voice quality may be compromised. Our previous study of 100 larynges of cadavers found that more tense and straighter vocal folds could be achieved using EAAL compared with other endoscopic glottis-widening procedures [43]. In cases of complete, permanent bilateral immobility, the preserved static components of voice production ensure a sociably acceptable voice with an adequate and stable airway at the same time.

Comparing the late postoperative voice of our patients to their original, ‘pre-thyreoidectomy’ voice quality would be an instructive study, but preoperative voice assessment is unfortunately not included in the presurgical protocol for thyroid/parathyroid surgery in Hungary. In our experience, we observed notable weakening of voice quality weeks after the onset of the BVFP. We conclude therefore that these early postoperative values cannot provide an exact basis for postoperative phoniatric analysis either. Nevertheless, in contrast to the conventional surgical approaches to BVFP (e.g., transverse cordotomy, partial/total CO_2_ laser arytenoidectomy, laterofixation via Lichtenberger’s needle carrier device), EAAL by ETGI provides better subjective and objective results in patients with BVFP simultaneously with higher peak inspiratory flows (Table 3), [2, 28–32].

**Table 3:** Long-term postoperative phoniatric parameters of different glottis enlarging procedures

	Aerodynamics	Acoustics				Perception				Dysphonia Index		
	MPT [s]	Pitch [Hz]	Jitt %	Shim %	HNR [dB]	VHI	G	R	B	DSI	FDI	PIF[l/s]
Physiological Values	> 15		< 1.04	< 3.81	> 20	0–120	0–3	0–3	0–3	(− 5)–(5)	0–3	
Pruzewicz et al. [29] 13 patients with laser arytenoidectomy (12 females, 1 male)												
Mean		238.00	2.30	7.00								
SD ( ±)	Not published											
Dursun et al. [30] 22 patients with transverse cordotomy (14 females, 8 males)												
Mean	7.30	184.00	1.75	7.01	12.45							
SD ( ±)	Not published											
Harnisch et al. [2] 10 patients with mainly transverse cordotomy (8 females, 2 males)												
Mean	4.6	202.60	5.02	24.93		55.00	2.00	1.00	2.00	− 5.60	2.16	1.61
SD ( ±)	2.7	39.60	5.46	9.47		19.00	1.00	1.00	1.00	6.27	0.50	0.49
Yilmaz et al. [31] 20 patients after endoscopic partial/total arytenoidectomy (15 females, 5 males)												
Mean	9/8	214/224		9.75/9.99	8.54/8.24*							
SD ( ±)	Not published											
Lawson et al. [32] 46 patients after subtotal arytenoidectomy/posterior cordectomy (28 females, 1 male)												
Mean	6.8/7.8											
SD ( ±)	2.6/1.6											
Nawka et al. [33] 36 patients after permanent transoral surgery (posterior cordotomy, laterofixation, partial arytenoidectomy) (30 females, 6 males)												
Mean	7.41		0.84				2.0	1.5	2.0	− 0.11		1.4
SD ( ±)	3.67		0.86							3.60		0.9

*MPT* maximum phonation time, *Jitt* Jitter, *Shim* Shimmer, *HNR* Harmonic to Noise Ratio, *VHI* Voice Handicap Index, *G* global, *R* roughness, *B* breathiness (from the GRBAS scale), *DSI* Dysphonia Severity Index, *FDI* Friedrich’s Dysphonia Index, *PIF* Peak Inspiratory Flow

*HNR was calculated as 10 log(1/NHR) [45]

In our experience, swallowing problems caused by unilateral EAAL are extremely rare. If present, they are temporary and occur in the early postoperative period only. The minimally invasive EAAL does not damage either the surgically treated or the contralateral vocal fold, and therefore can take advantage of the potential regeneration of the RLN (*Groups I–III*). This way the glottis is surgically opened, but it is able to close during phonation or swallowing processes. After the procedure, the interarytenoid region remains intact as well, which is also essential to safe, aspiration-free swallowing [6]. Furthermore, due to its nondestructive manner, the intervention does not hinder the experience-dependent plasticity of the central nervous system controlling swallowing [44].

### Limitations

This study has certain limitations. Although, the presented objective voice parameters admittedly allow to infer the physical properties of vibration of the vocal fold, laryngeal stroboscopy or high-speed video laryngoscopy may provide further value to the postoperative analysis of the more delicate vocal fold motions. Further, future research should include laryngeal electromyography to stratify the patients according to the severity of their nerve injuries. However, the applied quality of life questionnaire includes a question about swallowing, a specific swallowing-related questionnaire or fiberoptic endoscopic evaluation of swallowing could provide a more detailed evaluation of the functional results.

## Conclusion

The final outcome of BVFP occurs within several months after the onset of recurrent laryngeal nerve injury; thus, BVFP should not be considered a static condition. Accordingly, one of the foundations of the current study was the proper categorization of BVFP patients based on the recovery of vocal fold motions. EAAL—as a quasidynamic surgical solution—provides an immediate adequate airway with reliable long-term results regardless of the recovery of the abduction movements. The procedure which will likely not disturb potential neuroregeneration meets the most important phoniatric requirements because of its minimally invasive manner and guaranteed reversibility. Therefore, EAAL provides beneficial phoniatric outcomes of patients with transient palsy. In cases of bilateral and unilateral motion recovery, the objective voice parameters reached or approximated the physiological ranges. Even patients with exclusively adductive regeneration had only minor voice impairment. Further, EAAL ensured a socially acceptable voice quality and an adequate airway, even for patients with permanent BVFP.
